# Depletion of placental brain-derived neurotrophic factor (BDNF) is attributed to premature ovarian insufficiency (POI) in mice offspring

**DOI:** 10.1186/s13048-024-01467-4

**Published:** 2024-07-09

**Authors:** Bin Liu, Yongjie Liu, Shuman Li, Pingping Chen, Jun Zhang, Liping Feng

**Affiliations:** 1 Ministry of Education-Shanghai Key Laboratory of Children’s Environmental Health, School of Medicine, Xinhua Hospital, Shanghai Jiao Tong University, Shanghai, China; 2 Department of Reproduction, School of Medicine, Xinhua Hospital, Shanghai Jiao-Tong University, Shanghai, China; 3 Department of Obstetrics and Gynaecology, Duke University, Durham, NC USA

**Keywords:** BDNF, PGC, Follicles, Oocyte, Cre, RNAseq, POI

## Abstract

**Introduction:**

Premature ovarian insufficiency (POI) is one of the causes of female infertility. Unexplained POI is increasingly affecting women in their reproductive years. However, the etiology of POI is diverse and remains elusive. We and others have shown that brain-derived neurotrophic factor (BDNF) plays an important role in adult ovarian function. Here, we report on a novel role of BDNF in the Developmental Origins of POI.

**Methods:**

Placental BDNF knockout mice were created using CRISPR/CAS9. Homozygous knockout (cKO(HO)) mice didn’t survive, while heterozygous knockout (cKO(HE)) mice did. BDNF reduction in cKO(HE) mice was confirmed via immunohistochemistry and Western blots. Ovaries were collected from cKO(HE) mice at various ages, analyzing ovarian metrics, FSH expression, and litter sizes. In one-month-old mice, oocyte numbers were assessed using super-ovulation, and oocyte gene expression was analyzed with smart RNAseq. Ovaries of P7 mice were studied with SEM, and gene expression was confirmed with RT-qPCR. Alkaline phosphatase staining at E11.5 and immunofluorescence for cyclinD1 assessed germ cell number and cell proliferation.

**Results:**

cKO(HE) mice had decreased ovarian function and litter size in adulthood. They were insensitive to ovulation induction drugs manifested by lower oocyte release after superovulation in one-month-old cKO(HE) mice. The transcriptome and SEM results indicate that mitochondria-mediated cell death or aging might occur in cKO(HE) ovaries. Decreased placental BDNF led to diminished primordial germ cell proliferation at E11.5 and ovarian reserve which may underlie POI in adulthood.

**Conclusion:**

The current results showed decreased placental BDNF diminished primordial germ cell proliferation in female fetuses during pregnancy and POI in adulthood. Our findings can provide insights into understanding the underlying mechanisms of POI.

**Supplementary Information:**

The online version contains supplementary material available at 10.1186/s13048-024-01467-4.

## Introduction

Premature ovarian insufficiency (POI) is an important cause of women’s infertility that is defined as the inability of a couple to conceive after 12 months of trying [1] and infertility rates are rising [2]. The increase in the rate of infertility and childless women is mainly due to the increase in the age of first pregnancy or ovarian reserve function declines early [3, 4]. The ovarian reserve refers to the capacity of the ovary to provide eggs that are capable of fertilization resulting in a healthy and successful pregnancy. The ovarian reserve is mainly associated with a woman’s chronological age [5]. However, more and more women now have low ovarian reserve at young reproductive ages [6], which will lead to infertility in 1–5% of women under the age of 40 [7, 8]. The occurrence of POI is thought to be due to decreased ovarian reserve [9]. The causes of the majority of POI or decreased ovarian reserve are mysterious and remain to be elucidated.

In mammals, the oocytes originate from primordial germ cells (PGCs) [10]. In mice, PGCs first appear around embryonic day 6.5 (E6.5) and are identified as a cluster of about 40 cells at around E7.5 [11–13]. Then PGCs begin to migrate and proliferate until E13.5 [14–18]. That’s when the germ cell pool is established and becomes a foundation for the reproductive reserve in the female embryo [10]. There is now a finite supply of female germ cells that cannot be regenerated if lost or come up short [19, 20]. Once the proliferation of early PGCs is affected, the ovarian reserve after birth will be affected, which may also be the cause of some unexplained POI. Therefore, the intrauterine environment and the placenta of the pregnant mother are crucial to the establishment of ovarian reserve function.

Brain-derived neurotrophic Factor (BDNF) is a member of the neurotrophin family of growth factors that are found in the brain and the periphery. BDNF mainly activates TrkB tyrosine kinase receptor. The activation of the BDNF-TrkB pathway plays a significant role in the survival, development, and functionality of nerve cells. It promotes the survival and development of neurons, enhances synaptic plasticity, regulates interneuronal communication, and neurogenesis [21]. Recently, it was reported that BDNF plays an important role in ovarian functions [22, 23]. BDNF has been reported to promote the differentiation of embryonic stem cells [24, 25]. However, the effect of BDNF on the generation and proliferation of PGCs has not been reported. During pregnancy, the origin of fetal BDNF is thought to be the placenta which can generate BDNF and transfer maternal BDNF to the fetal compartment [26].

In this study, we established a placental-specific BDNF knockout mouse model to investigate the role of placental BDNF in the development of ovarian reserve during fetal development and ovarian function later in life. Our study demonstrated that decreased placental BDNF expression during gestation leads to POI in offspring by affecting the proliferation of early PGCs. Our findings uncovered a Developmental Origin of some unexplained POI that can be intervened.

## Materials and methods

### Animals

Mice were housed in a specific pathogen-free (SPF) and climate-controlled facility at constant room temperature (22–25 °C) under a 12 h light: 12 h dark cycle, with humidity control (60 ± 10%). Mice were provided with basic mouse chow and distilled water ad libitum at Shanghai Jiao-Tong University School of Medicine, Xinhua Hospital Laboratory Animal Center. Animal care practices were followed by the ethical guidelines of the International Association for the Study of Pain regarding the use of laboratory animals and were reviewed and approved by the Animal Committee of Shanghai Jiao-Tong University School of Medicine, Xinhua Hospital Laboratory Animal Centre, Shanghai, China.

*Elf5-Cre* transgenic mice were the gift of Dr. Haibing Wang from Xiamen University [27]. Mice carrying *Elf5-Cre*^+^*BDNF*^*floxed*^ gene or [cKO(HE)] mice were produced by crossing *Elf5-Cre* males with *BDNF*^*loxP/loxP*^ females (generated by Shanghai Model Organisms using CRISPR-Cas9 technology derived from C57BL/6 mice.). In theory, 50% of F1 pups are *Elf5-Cre*^+^*BDNF*^*floxed*^ [cKO(HE)] and 50% of F1 pups are *Elf5-Cre*^*−*^*BDNF*^*loxP*^ (controls) (Supplementary Fig. 1).

### Genotyping

*Elf5-Cre*^+^*BDNF*^*floxed*^ [cKO(HE)] animals were genotyped by PCR using tail genomic DNA and two sets of specific oligodeoxynucleotide primers. The primer pair used to genotype transgenic mice that carry the BDNF gene recognizes a DNA segment within exon 9 in the BDNF gene. The size of the expected amplicon of the loxp allele is 363 bp. The size of the expected amplicon of floxed allele is 311 bp. (Forward primer: 5’-TGCTAAAGCGGGAGGAAGTG-3’, Reverse primer: 5’-GGAACTGTGGGAAGGAAGCA -3’). The size of the expected amplicon of *Elf5-Cre*^+^ or *Elf5-Cre*^*−*^ is 200 bp and 450 bp, respectively. (Primer 1: 5’-TAATGGTGCAACGGGTCCTC-3’, Primer 2: 5’-GAATTCTGGAGCGGGTTGAC-3’, Primer 3: 5’- TTCTTGCGAACCTCATCACTC-3’).

### Ovulation induction and oocyte collection

Oocyte superovulation was induced by an intraperitoneal (IP) injection of Pregnant Mare Serum Gonadotrophin (PMSG, 10 IU) for 48 h followed by an IP injection of hCG (10 IU) for 14 h. Mice were then sacrificed by cervical dislocation, and oviducts were removed. Oocyte-cumulus complexes were collected from the oviduct ampulla and digested in 80 IU/mL hyaluronidase solution (IrvineScientific, cat#: 90,101) before counting oocytes.

### Ovarian and oocyte smart RNAseq and data analysis

Collect the ovarian tissue from P7 mice and carefully remove the surrounding fallopian tubes and ovarian membrane under a stereomicroscope. Subsequently, the intact ovarian tissue was transferred into a lysis solution for smart RNAseq.

The eggs that were retrieved from one-month-old mice after superovulation were blown and washed repeatedly in an acidic benchtop solution to remove the zona pellucida, then placed in a lysis buffer for smart RNAseq.

Both ovaries and oocytes were subjected to transcriptome sequencing using smart RNAseq as follows. Full-length cell RNA-seq libraries were generated following the Smart-seq2 protocol [28, 29]. In brief, the cell was rapidly isolated into PCR tubes containing lysis buffer. Reverse transcription was performed using SMARTScribe Reverse Transcriptase (Takara) along with oligo-dT30VN, template-switching oligonucleotides, and betaine. The resulting cDNA was amplified using SeqAmp DNA Polymerase (Takara), PCR primers, and 18 amplification cycles. After purification using Agencourt Ampure XP beads (Beckmann Coulter), the size distribution and quantity of the amplified product were assessed using a High Sensitivity DNA Kit on a Bioanalyzer (Agilent Technologies). Subsequently, 50 ng of the amplified cDNA was fragmented using Covaris® M220 (Covaris). The fragmented cDNA was then subjected to end-repair, A-tailing, and ligation of custom KAPA Dual-indexed Adapters using the KAPA HyperPrep Kit (Roche). The resulting products were purified twice using Agencourt Ampure XP beads, amplified for 15 cycles, and quantified again using a Bioanalyzer High Sensitivity DNA Kit. Finally, the library was subjected to Illumina sequencing using paired-end 2 × 150 sequencing mode.

Gene expression levels were quantified using FPKM (fragments per kilobase of exon per million fragments mapped) calculated by StringTie v1.3.4d with default parameters [30]. Differential gene expression analysis was performed using the R package edgeR v3.24.2 [31]. To assess the significance of the observed differences, adjusted *P*-values were calculated using the false discovery rate (FDR) control method for multiple testing. Only genes with an adjusted *P*-value < 0.05 and absolute log2 fold change (|log2FC|) ≥ 1 were considered for subsequent analysis.

The gene annotation file was obtained from the Ensembl genome browser 96 databases (http://www.ensembl.org/index.html). To annotate genes with Gene Ontology (GO) terms [32, 33], and KEGG pathways [34], we utilized the ClusterProfiler v3.4.4 [35] R package. Functional enrichment analysis for both GO and KEGG was conducted using ClusterProfiler.

### Reverse transcription-quantitative polymerase chain reaction (RT-qPCR)

Reverse transcription PCR (RT-PCR) was performed according to the protocol described previously using the HiScript 1st Strand cDNA Synthesis Kit (Jizhen Biology), with the following procedure: 25 ◦C for 5 min, 50 ◦C for 15 min and 85 ◦C for 5 min [36]. Quantitative polymerase chain reaction (PCR) was conducted in triplicate using the following components: 2 μl of cDNA, 5 μl of PowerUp™ SYBR™ Green Master Mix (ThermoFisher Scientific), 1 μl of primer working solution and ddH_2_O to 10ul. Thermal cycling conditions consisted of an initial step at 95 °C for 5 min, followed by 44 cycles of 96 °C for 10 s and 60 °C for 30 s. The amplifications were monitored using the Applied Biosystems 7500 Fast Real-Time PCR System (ThermoFisher). The obtained results were normalized to the expression of the housekeeping gene *Gapdh*, and relative gene expression was analyzed using the 2^−ΔΔCt^ method. Primers used in Supplementary Table 1.

### SEM

Ovaries were fixed in 2.5% glutaraldehyde at room temperature for 2 -4 h. The ovaries were washed with PBS (PH7.4) three times for 15 min each. The ovaries were then placed in 1% osmic acid for 2 h. The ovaries were washed with PBS three times for 15 min each. Then dehydrated with 50%, 70%, 80%, 90%, and 100% ethanol for 15 min each. The ovaries were transferred into 1:1 acetone: resin for 2–4 h, 1:2 acetone: resin overnight, and acetone in a 37℃ oven overnight, then in a 60℃ oven for 48 h. The ovaries were sectioned 60-80 nm by ultra-thin microtome (Leica UC7). Photographs were taken via SEM (HT7700, HITACHI).

### Serum collection

Under isoflurane anesthesia, blood samples were collected from the auricula dextra of all mice into 1.5 mL microcentrifuge tubes and kept on ice for 2 h for clotting. Serum was obtained via centrifugation at 3500 × rpm for 15 min and stored at -80 °C for further analysis.

### Histological staining

Ovaries were collected from all mice, fixed in 4% paraformaldehyde (PFA) for at least 24 h at room temperature, and paraffin-embedded. The embedded ovaries were sectioned consecutively into five sections of 5 μm thickness. Hematoxylin and eosin (H&E) staining of all five sections was used to determine the number of follicles at various stages of development by systematic counts.

### Alkaline phosphatase staining

Pregnant mice at E11.5 were euthanized, and embryo tails were collected for genotyping. The embryos were then fixed in 4% PFA overnight at 4 °C and washed three times with PBS. The genital ridges were dissected carefully and embedded in paraffin. The paraffin blocks were cut into 5 μm thickness slides. Paraffin slides were dewaxed, kept in tap water, and incubated at 37 °C for 4 h. The slides were washed with distilled water twice and treated with Duolink in Situ Detection Reagents for 5 min. The slides were then washed with distilled water 3 ~ 5 times, treated with an alkaline phosphatase dye solution for 30–60 s, and then washed in running water for 5 min. The slides were stained in hematoxylin solution for 3 ~ 5 min and then washed in running water. 1% HCl solution was used to differentiate for 3 s followed by rinsing in tap water and then in hydroxide for 5 s. Finally, the slides were rehydrated and mounted with neutral resin after washing them in running water. Slides were observed under a microscope and images were taken.

### ELISA for serum and ovarian tissue analysis

BDNF protein levels in serum and ovaries were quantified using a Mouse BDNF ELISA kit (MULTI SCIENCES, Cat#: EK2127-01) following the manufacturer’s instructions. The ovarian tissue level of FSH was determined by a Mouse FSH ELISA kit (SAB, Cat#: EK14090) following the manufacturer’s instructions. The optical density (OD) of ELISA results was measured by a microplate reader (Thermo, Multiskan Go1510) at 450 nm and the concentrations were calculated using the standard curve.

### Western blot

Placentas and ovaries were incubated on ice with lysis buffer (Beyotime, Cat#: P0013C) supplemented with PMSF, a proteinase inhibitor. After homogenization, the lysates were centrifuged at 14,000 rpm at 4 °C for 5 min. The supernatant was collected and stored at -80 °C. Protein concentrations of the supernatants were measured using a BCA protein assay (Beyotime, Cat#: P0011). The protein samples were denatured and separated on 12% SDS-PAGE gels and then transferred to PVDF membranes (Millipore) by electrical transfer. PVDF membranes were then blocked using Protein Free Rapid Blocking Buffer (Beyotime, Cat#: P0575) for 15 min at room temperature and then incubated overnight at 4 °C with primary antibodies: Rabbit anti-mouse BDNF (1:200 dilution, Merck, Cat#: SAB2108004).

### Statistical analysis

All results are presented as mean ± SD. Data were assessed using GraphPad Prism 8.0.2 (La Jolla, CA) and SPSS 26.0 software (SPSS, Inc., Chicago, IL, USA). Statistical analysis was performed using an unpaired Student’s t-test for two comparisons or one-way ANOVA for multiple comparisons. A *p*-value less than 0.05 was statistically significant.

## Results

### Establish a placental-specific BDNF knockout mouse model (cKO mouse)

**Fig. 1:**
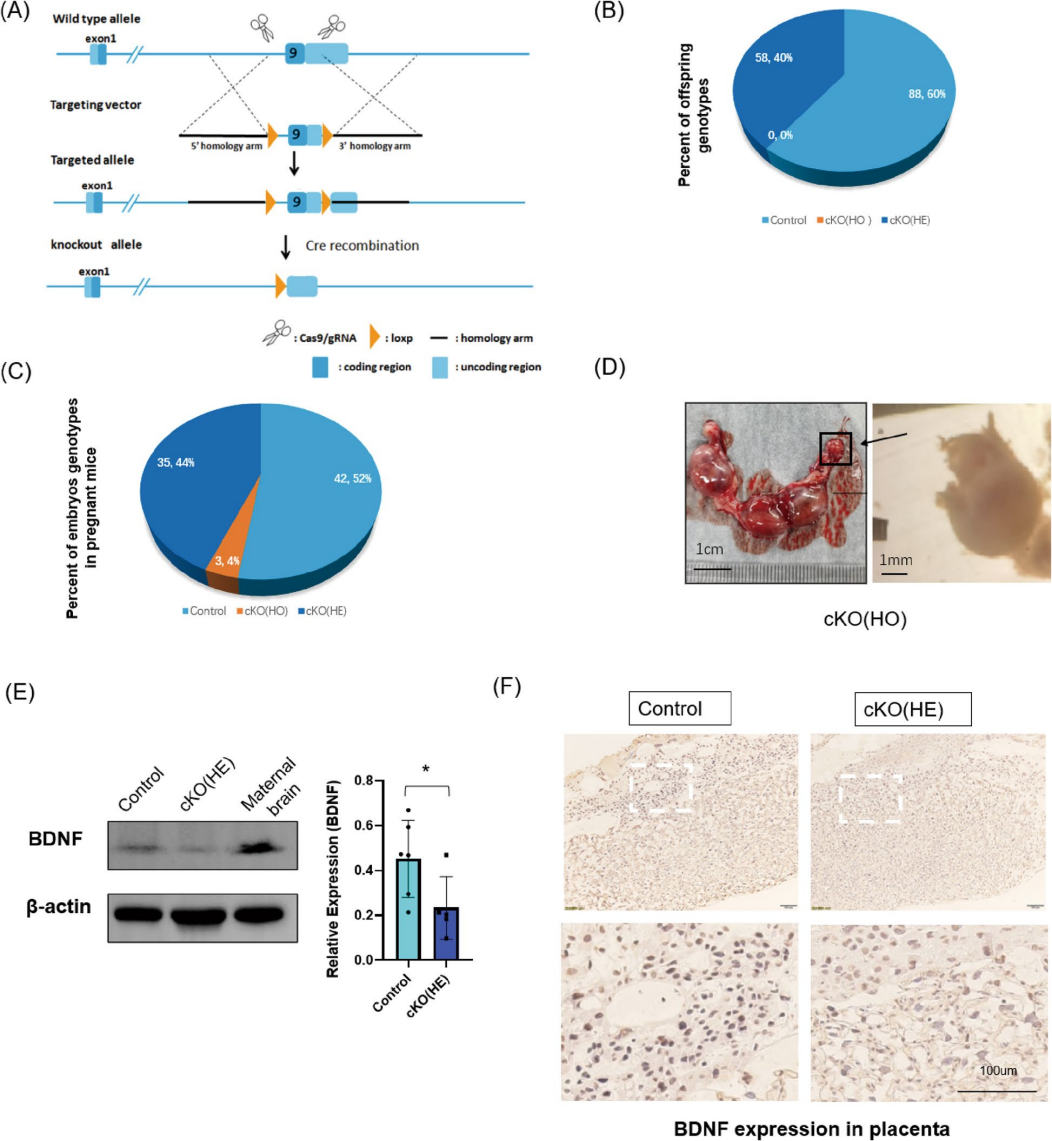
Establishment of a placental conditional BDNF knockout mouse model. **A** the cre-loxP system for generating a placental conditional BDNF knockout mouse model. **B** number and percent of offspring with different genotypes (*n* = 146). **C** number and percent of embryos with different genotypes (*n* = 80). **D** abnormal cKO(HO) embryos (*n* = 3). **E** a representative Western blot image (5 controls and 4 cKO(HE)s) placenta and maternal brain tissues) and the densitometry of BDNF bands in Western blot after normalization to β-actin. **F** immunohistochemical staining of BDNF in the placenta (*n* = 3). Data are presented as mean ± SD. * indicates *P* < 0.05. NS indicates not significant

Cre-loxP technology was used to generate cKO mice (Fig. 1A). Elf5-cre mice (*Elf5-Cre*^+^ mice) were kindly given by Dr. Haibin Wang for trophoblast-specific gene manipulation [27]. LoxP BDNF mice (*BDNF*^*loxP/loxP*^ mice) were purchased from Shanghai Model Organisms. *Elf5-Cre*^+^ mice were bred with *BDNF*^*loxP/loxP*^ mice to generate heterozygous placental-specific BDNF knockout mice [cKO(HE) mice]. To produce homozygous knockout mice [cKO(HO) mice], cKO(HE) male mice were bred with *BDNF*^*loxP/loxP*^ or cKO(HE) females. However, no alive cKO(HO) mice were born (Fig. 1B). Abnormal cKO(HO) embryo masses (early absorptions) were found at necropsy at E18.5 (Fig. 1C-D). Thus, cKO(HE) mice were used for this study as cKO(HO) appeared to be lethal. To determine the effectiveness and specificity of elf5-Cre to reduce BDNF expression in the placenta, we measured BDNF protein in the placenta and maternal brain. We demonstrated that BDNF protein expression was significantly reduced in cKO(HE) placentae by Western Blot (Fig. 1E) and immunohistochemical staining (Fig. 1F) compared to the controls. In maternal brains, BDNF protein expression was not changed among groups.

### The ovarian function and reproduction were decreased in cKO(HE) female mice

**Fig. 2:**
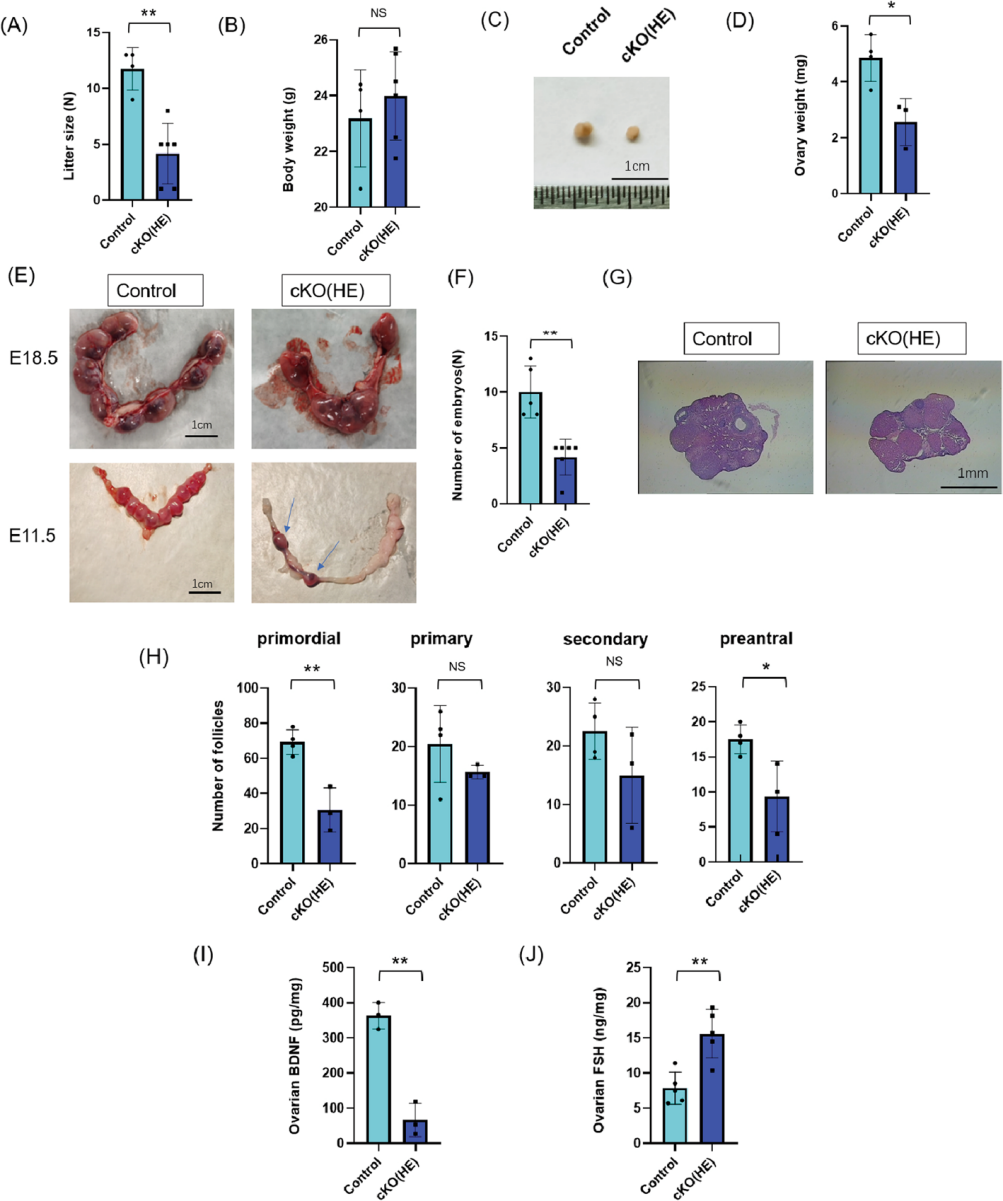
Decreased ovarian function and reproduction in four-month-old cKO(HE) female mice. **A** litter size and (**B**) pup weight in the controls (*n* = 4) and cKO(HE) mice (*n* = 6). **C**-**D** ovarian volume and ovarian mass in the control (*n* = 4) and cKO(HE) (*n* = 3) mice. **E**–**F** gravid uterus at E18.5 and E11.5 in the control (*n* = 5) and cKO(HE) (*n* = 6) mice. **G.** representative H&E-stained sections of ovaries (*n* = 3). **H** the number of primordial, primary, second, and preantral follicles in controls (*n* = 3) and cKO(HE) (*n* = 3) mice. **I** BDNF protein expression in ovaries was measured by ELISA (*n* = 3). **J** FSH protein expression in ovaries was measured by ELISA in the controls (*n* = 5) and cKO(HE) (*n* = 5) mice. Data are presented as mean ± SD. NS indicates not significant. * Indicates *P* < 0.05. ** indicates *P* < 0.01

We first noticed that four-month-old cKO(HE) female mice had abnormal litters. Specifically, the number of pups significantly decreased although there were no significant differences in pup weight in litters of four-month-old cKO(HE) mice compared to litters of control mice (Fig. 2A-B). The ovarian weight and volume were significantly lower in four-month-old cKO(HE) mice compared to the control mice (Fig. 2C-D). There are visible gaps and absorbed embryos in the gravid uterus of cKO(HE) mice at E11.5 and E18.5 (Fig. 2E). The number of follicles is a predictor of oocyte production. H&E staining was used to examine the ovarian follicle development (Fig. 2F). The numbers of primordial and preantral follicles in four-month-old cKO(HE) mice were significantly lower than those in the control mice, but the numbers of primary and secondary follicles were not different (Fig. 2G). BDNF protein expression in ovaries significantly decreased in four-month-old cKO(HE) mice compared with the control mice (Fig. 2H). FSH expression in ovaries significantly increased in four-month-old cKO(HE) mice compared to the control mice (Fig. 2I).

**Fig. 3:**
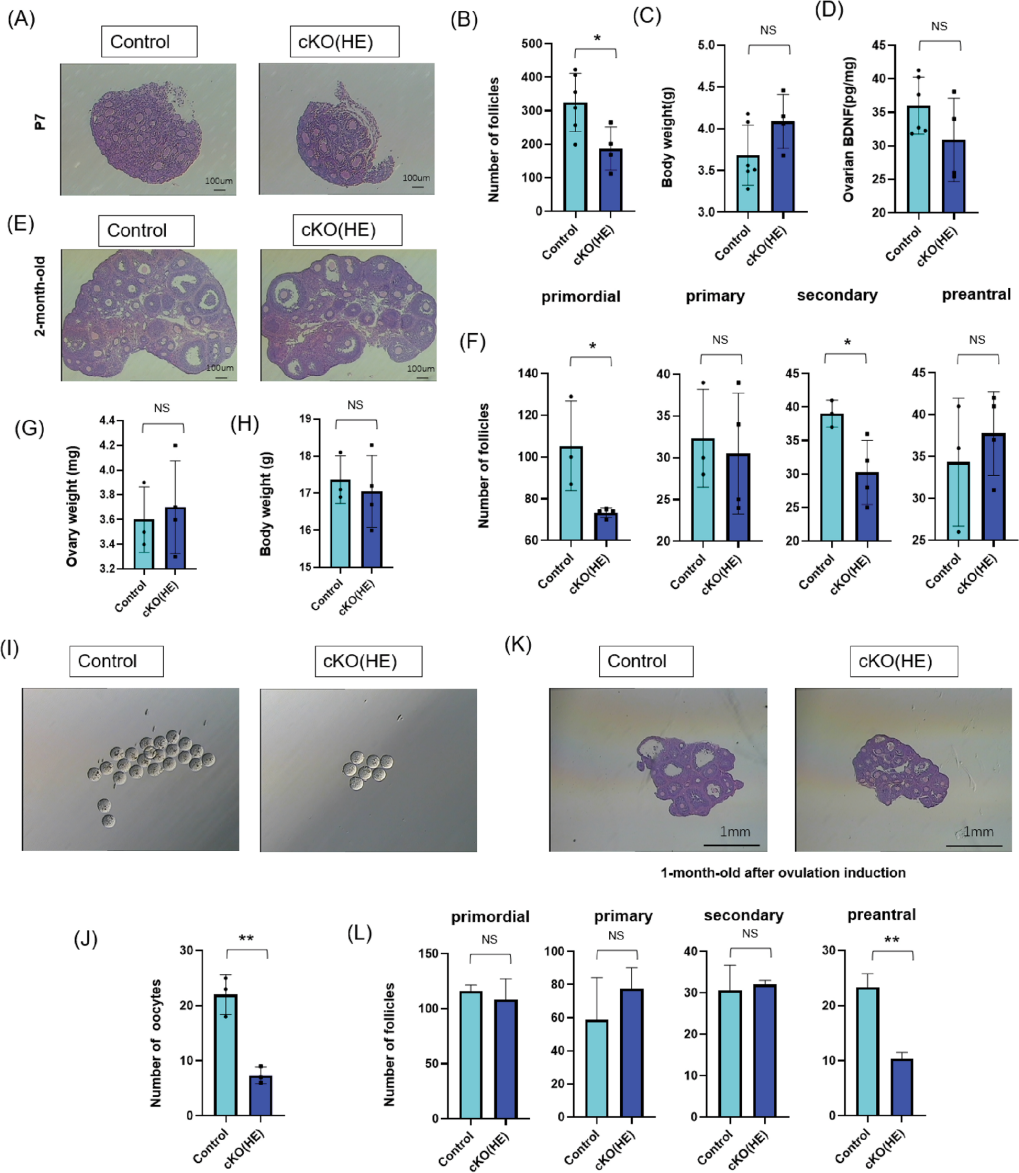
Decreased ovarian function in young cKO(HE) female mice. **A** representative H&E-stained sections of ovaries in postnatal-day-7-old pups (P7) in the controls (*n* = 6) and postnatal-day-7-old cKO(HE) (*n* = 4) mice. **B** the number of follicles in ovaries in the controls (*n* = 6) and postnatal-day-7-old cKO(HE) (*n* = 4) mice. **C** body weight of the controls (*n* = 6) and postnatal-day-7-old cKO(HE) (*n* = 4) mice. **D** BDNF expression in ovarian of postnatal-day-7-old in the controls (*n* = 6) and postnatal-day-7-old cKO(HE) (*n* = 4) mice. **E** representative H&E-stained sections of ovaries in two-month-old mice in the controls (*n* = 3) and two-month-old cKO(HE) (*n* = 4) mice. **F** the number of primordial, primary, secondary, and preantral follicles in the controls (*n* = 3) and two-month-old cKO(HE) (*n* = 4) mice. **G**-**H** ovary and body weight in controls (*n* = 3) and two-month-old cKO(HE) (*n* = 4) mice. **I** Representative images of eggs of one-month-old mice (*n* = 3). **J** the number of eggs in the controls (*n* = 3) and one-month-old cKO(HE) (*n* = 3) mice. **K** representative H&E-stained sections of ovaries after ovulation induction in one-month-old mice (*n* = 3). **L** the number of primordial, primary, second, and preantral follicles in controls (*n* = 3) and one-month-old cKO(HE) (*n* = 3) mice after ovulation induction. Data are presented as mean ± SD. NS indicates not significant. * Indicates *P* < 0.05. ** indicates *P* < 0.01

We then examined the ovarian function in younger cKO(HE) mice. We found that the number of follicles significantly decreased in postnatal-day-7-old (P7) cKO(HE) pups compared with the controls while the body weight of pups had no difference (Fig. 3A-C). The BDNF protein expression in ovaries was not different (Fig. 3D). In the two-month-old mice, the number of primordial and secondary follicles in cKO(HE) mice was significantly lower than those in the controls, while the numbers of primary and preantral follicles were not different comparing to the controls (Fig. 3E-F). The ovary and body weight of these mice were not different (Fig. 3G-H). One-month-old female mice are the best recipients for superovulation and harvesting eggs [37]. In one-month-old cKO(HE) mice, the number of eggs significantly decreased compared with the controls (Fig. 3I). H&E staining was used to examine the ovarian follicle after superovulation (Fig. 3J). There is no difference in primordial, primary, and secondary follicles. However, the number of preantral follicles in one-month-old cKO(HE) mice was lower than those in the controls after superovulation (Fig. 3K-L). These results indicate that ovaries in cKO(HE) mice are not sensitive to superovulation induction drugs, pregnant mare serum gonadotropin (PMSG), and human chorionic gonadotropin (hCG). Gonadotropins such as PMSG and hCG have follicle-stimulating activity and induce oocyte maturation, leading to ovulation and the production of mature eggs [38, 39]. The decrease in the number of mature eggs produced in young cKO(HE) mice after superovulation indicates that the number of available follicles for recruitment was reduced or cells in follicles including oocytes, granulosa cells, and theca cells are less responsive to gonadotrophin.

In summary, we observed premature ovarian failure (POI) phenotype in cKO(HE) mice.

### Transcriptome analysis identified differentially expressed genes (DEGs) in ovaries

**Fig. 4:**
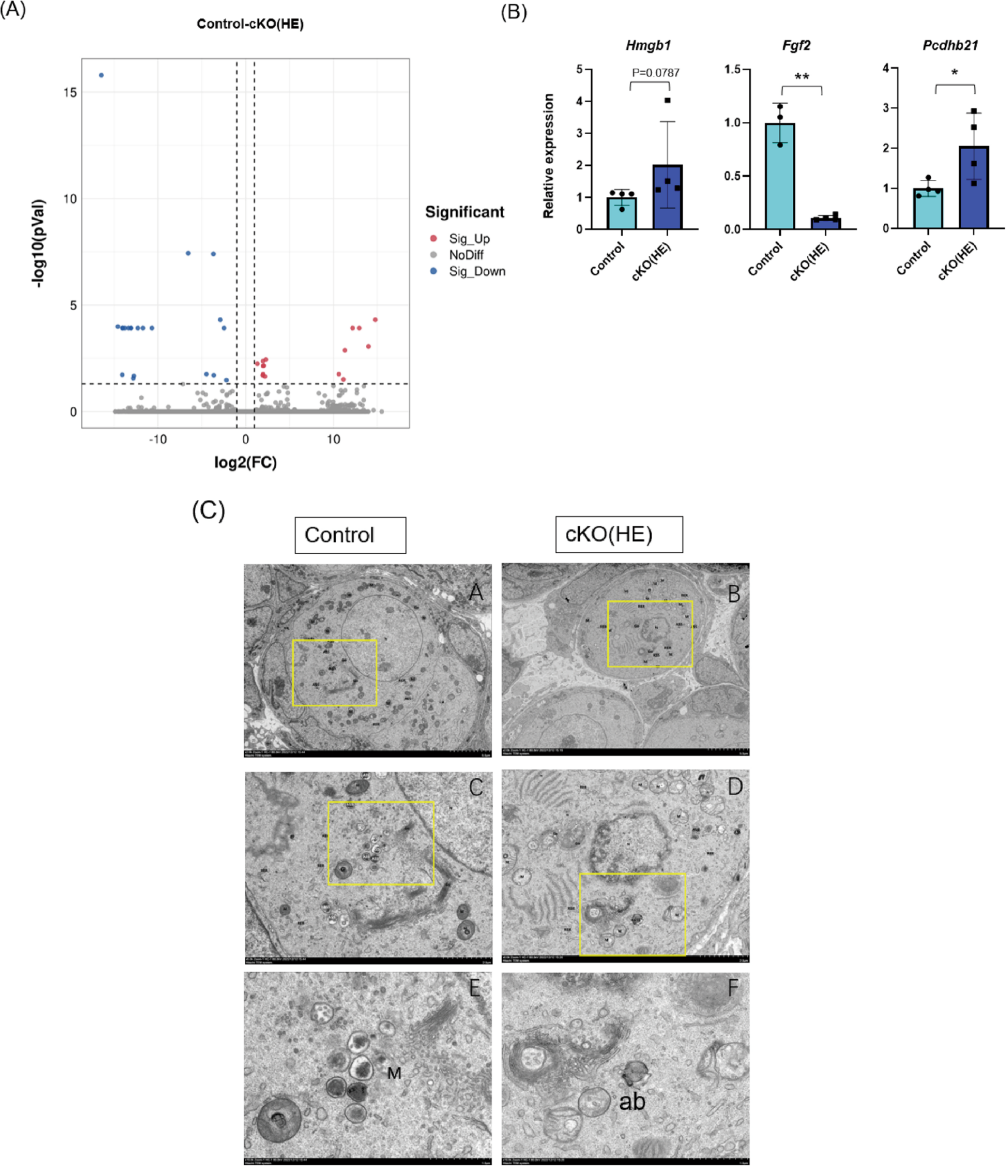
Transcriptome, real-time qPCR, and electron microscope analysis of the ovaries of postnatal-day-7-old (P7) mice. **A** volcano plot showing the differentially expressed genes (DEGs) in the P7 cKO(HE) ovaries (*n* = 3) and the controls (*n* = 3). Red dots represent the up-regulated genes in P7 cKO(HE) ovaries and blue dots represent the down-regulated genes in the P7 cKO(HE) ovaries. **B** RT-qPCR result (*n* = 4). **C** mitochondrial morphology under an electron microscope in ovaries (*n* = 3) (N: Nucleus; M: Mitochondrial; SER: Smooth endoplasmic reticulum; RER: Rough endoplasmic reticulum; Go: Golgi apparatus; ASS: Autolysosome; ab: apoptotic body)

As shown in the volcano plot, 15 genes were significantly upregulated and 21 genes were significantly downregulated in ovaries harvested from P7 cKO(HE) mice compared to the controls. (Fig. 4A). Three DEGs with known functions were selected for RT-qPCR validation (Supplemental Table 2). RT-qPCR results confirmed that the gene expression of fibroblast growth factor 2 (*fgf2)* was significantly downregulated, the gene expression of protocadherin beta 21 (*pcdhb21, p* < 0.05), and high mobility group box 1 (*hmgb1)* was up-regulated (*p* = 0.05) but not statistically significant in the cKO(HE) ovaries compared with the controls (Fig. 4B). These genes have been shown to mediate mitochondrial function and apoptosis [40]. There is increasing evidence suggesting that mitochondrial dysfunction plays a key role in ovarian aging [41, 42]. Thus, we further examined mitochondria in the ovaries of P7 pups using a Scanning Electron Microscope (SEM). SEM images revealed that the number of mitochondria was reduced and the average volume of mitochondria (swollen mitochondria) was increased in P7 cKO(HE) oocytes compared with the controls. Apoptotic bodies were also observed in cKO(HE) oocytes (Fig. 4C). The combination of the transcriptome and SEM results indicate that mitochondria-mediated cell death or aging might occur in cKO(HE) ovaries.

### Transcriptome analysis of oocytes harvested from one-month-old mice showed an aged gene profile in cKO(HE) oocytes compared to controls

**Fig. 5:**
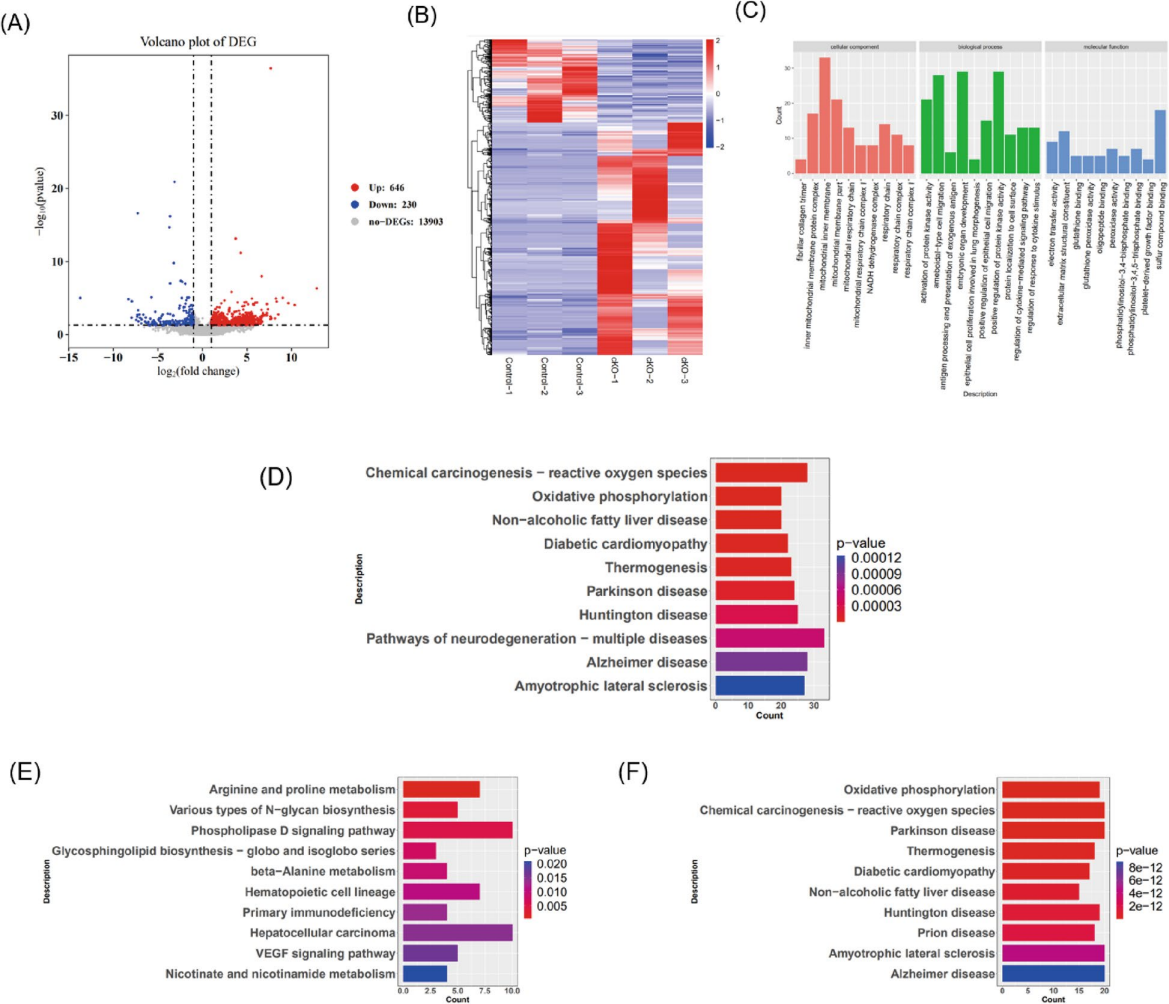
Transcriptome analysis of oocytes in one-month-old mice. **a** volcano plot showing the differentially expressed genes (DEGs) in oocytes in one-month-old cKO(HE) mice (*n* = 3) and the controls (*n* = 3). Red dots represent the up-regulated genes and blue dots represent the down-regulated genes in the cKO(HE) oocytes compared to the controls. **b** Heat map, red color represents higher expression, and blue color represents lower expression. **c** GO term analysis for DEGs. **d** KEGG pathway classification of DEGs. **e** KEGG pathway classification of up-regulated DEGs. **f** KEGG pathway classification of down-regulated DEGs

The transcriptome profile undergoes dramatic changes during oocyte aging. The oocytes are large and rich in RNA, which provides an advantage in RNA-seq (scRNAseq) studies. As shown in the volcano plot and heatmap, 646 genes were significantly upregulated and 230 genes were significantly downregulated in cKO(HE) oocytes compared to the controls (Fig. 5A-B). GO enrichment analysis indicated that mitochondrial inner membrane, embryonic organ development, and positive regulation of protein kinase activity were significantly enriched (Fig. 5C). KEGG pathway classification indicated that DEGs associated with chemical carcinogenesis − reactive oxygen species, thermogenesis, and oxidative phosphorylation signal pathways (Fig. 5D). The top 10 DEGs ranked according to *p*-values include *rpl37rt*, *gm4350*, *pcna-ps2*, *pakap*, *gm17535*, *pla2g4e, gm10800, duox2, gm10722, lrrc14, ugcg, gm37915, uqcr11*, *uba52*, *rpl41*, *ndufa11*, *gm1673*, *gm10076*, *samhd1* and *gm6166* (Supplementary Table 3). KEGG pathway enrichment analyses demonstrated that up-regulated DEGs were enriched in arginine and proline metabolism signals (Fig. 5E). And down-regulated DEGs were enriched in oxidative phosphorylation (Fig. 5F). The top 5 genes in arginine and proline metabolism pathway are *azin2*, *arg1*, *aoc1*, *prodh*, and *smox*. The top 5 genes in the oxidative phosphorylation pathway are *uqcr11*, *ndufs8*, *cox11*, *ndufa7*, and *ndufa11* (Supplementary Table 4). Increased arginine metabolism may result in the accumulation of methylarginines which has been shown to negatively impact IVF outcomes [43, 44]. Reduced mitochondrial metabolism and oxidative phosphorylation indicate that the oocytes are aging [45]. When oxidative phosphorylation is downregulated, it can lead to decreased ATP production, increased oxidative stress, and impaired cellular function [46, 47]. Increased oxidative stress can lead to cell damage, inflammation, and cellular aging. A study found that some genes associated with oxidative stress were significantly upregulated in the oocytes of older women, while they were downregulated in the oocytes of younger women [48, 49].

### The number and cell proliferation biomarkers of PGCs decreased in cKO(HE) embryos

**Fig. 6:**
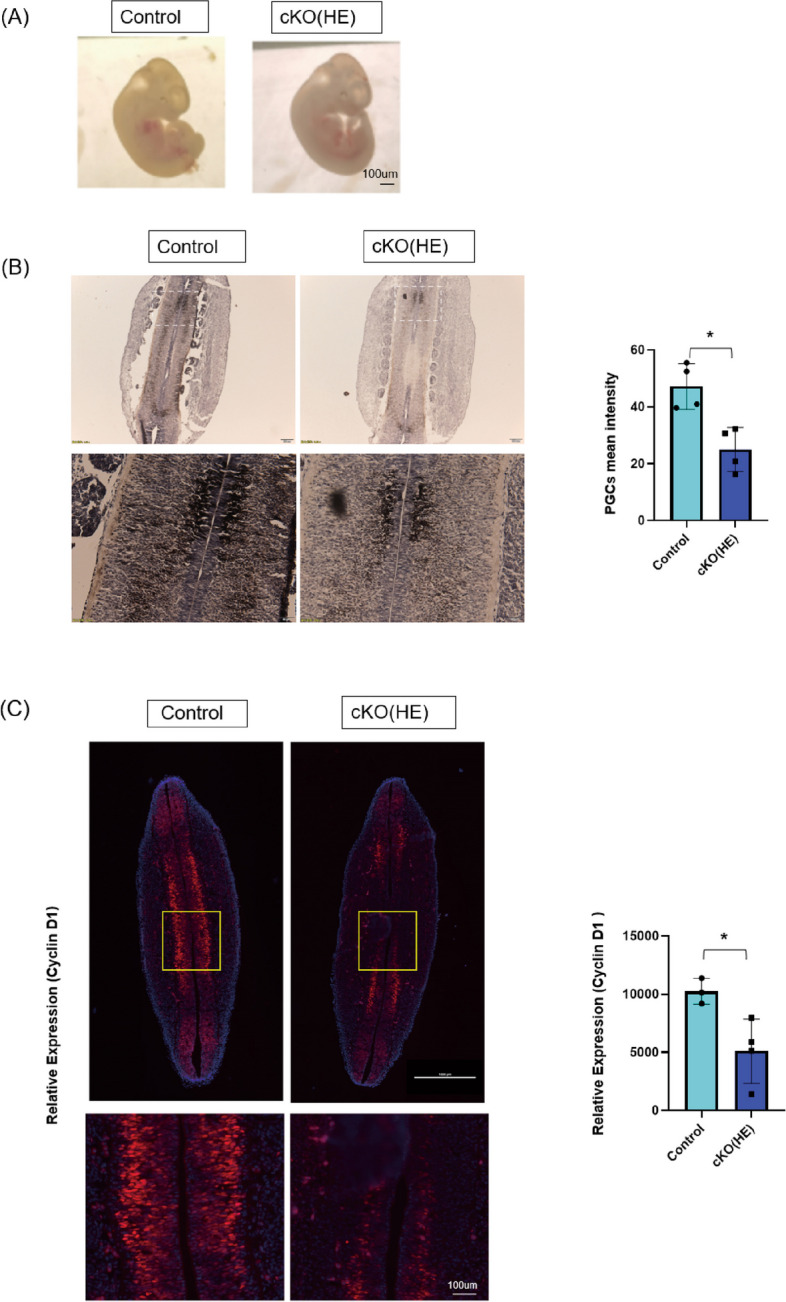
The number of PGCs and the expression of Cyclin D1, a cell proliferation biomarker. **A** the morphology of embryos at E11.5 (*n* = 3). **B** alkaline phosphatase staining of genital ridges and PGC counts at E11.5. The brown spots indicate PGCs cKO(HE) mice (*n* = 3) and the controls (*n* = 3). **C** Representative immunofluorescent staining images for Cyclin D1 and the quantitive analysis of Cyclin D1 expression in embryos’ genital ridges cKO(HE) mice (*n* = 4) and the controls (*n* = 3)

The ovary BDNF levels of postnatal-day-7-old pups were not different between cKO(HE) mice and the controls. Thus, we suspect the POI phenotypes in cKO(HE) offspring originated from embryo development. We did not observe noticeable differences in the morphology of embryos at E11.5 (Fig. 6A). Intriguingly, alkaline phosphatase staining demonstrated that the number of PGCs significantly decreased in cKO(HE) embryos compared with the controls (Fig. 6B). As PGCs are highly proliferative between E7.5 and E13.5 to establish the ovarian reserve, we suspected that PGCs cell proliferation was inhibited in cKO(HE) mice. Previous studies reported that BDNF regulates cell proliferation through Cyclin D1 [50, 51]. Here, we report that the expression of Cyclin D1 significantly decreased in cKO(HE) embryos’ genital ridges compared with the controls (Fig. 6C). Overall, we demonstrated that the PGCs proliferation and PGCs pool were negatively affected by BDNF knockdown in the placenta in cKO(HE) embryos. The diminished ovarian reserve during embryonic development may underlie the observed POI in cKO(HE) offspring.

### Placenta BDNF-PGCs-POI- axis

The reduction of BDNF in the placenta seems to have a significant impact on the development of the embryos’ genital ridges. The decreased Cyclin D1 expression in these ridges suggests a potential disruption in cell cycle regulation, which can affect the proliferation of PGCs. This reduction in PGC number can have long-term consequences, as it may lead to POI in adulthood. Furthermore, the observed increase in *hmgb1* expression, along with decreased *fgf2* expression and mitochondrial dysfunction in P7 cKO(HE) ovaries. Dysregulation of mitochondrial function can impact various cellular processes, including energy production and signaling pathways, which are essential for ovarian health and function. Taken together, these findings suggest a complex interplay between placental BDNF levels, Cyclin D1 expression, PGC development, and ovarian aging. Understanding these molecular mechanisms can provide valuable insights into the pathogenesis of POI and age-related ovarian decline, highlighting the importance of placental health, cellular signaling pathways, and mitochondrial function in reproductive biology.

**Fig. 7:**
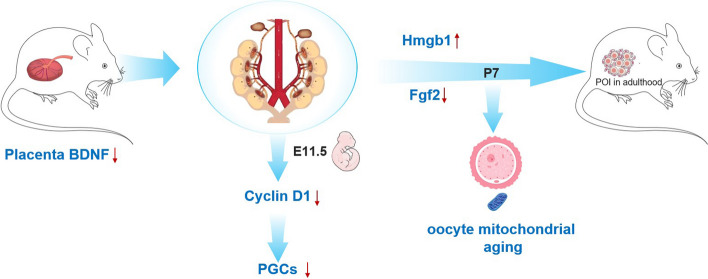
The schematic mechanism. Placenta BDNF-PGCs-POI- axis

Thus, the reduction of placenta BDNF leads to a reduced Cyclin D1 expression in embryos’ genital ridges and a reduced number of PGCs. which ultimately leads to POI in adulthood. The increased *hmgb1* expression and decreased *fgf2* expression and dysregulated mitochondrial function in P7 cKO(HE) ovaries indicate that ovarian aging in these mice may be mediated by mitochondria (Fig. 7).

## Discussion

BDNF has been shown to play a critical role in ovarian function in adults. In this study, we have uncovered the role of placental BDNF during pregnancy in the developmental origin of POI in adulthood. When BDNF is genetically ablated partially in the placenta, the primordial germ cell proliferation is impeded, and the establishment of a germ cell pool or reproductive reserve is insufficient. Consequently, ovarian function and reproduction were remarkably decreased in cKO(HE) female mice at their reproductive age. Transcriptome analysis and SEM examination indicated that the oocytes were prematurely aged in one-month-old cKO(HE) mice.

During embryonic development, the placenta has a significant impact on the growth and development of fetuses [52] and their long-term health [53]. Early embryo development is a process of rapid cell proliferation and differentiation. During this process, the placenta not only provides oxygen and nutrients but also produces numerous hormones and growth factors for regulating embryo development. Placental developmental and functional defects are threats to pregnancy and affect the short- and long-term development of the offspring [54, 55]. Detection of neurotrophins and their receptors in human and mouse placenta and fetal tissues has been reported [56, 57]. BDNF is expressed in the mouse placenta throughout gestation [57]. Thus, the transport and synthesis of BDNF in the placenta may be the major source of fetal BDNF during gestation [26, 58, 59]. The source of BDNF in the fetal compartment during human pregnancy may be endogenous fetal synthesis, maternal passage, and placental production [60]. Studies in humans demonstrated that BDNF dysregulation in the placenta resulted in abnormal fetal growth and is associated with the later development of adult disease [61–63]. BDNF and its receptor, tyrosine kinase B (TrkB), were detected in oocytes and granulosa cells (GCs) of human and mouse fetuses. BDNF and neurotrophin 4 (NT4) have been demonstrated previously to be important during fetal ovary development [64–68]. How BDNF promotes fetal ovarian development and ovarian functions later in life is not clear. Our finding suggests that decreased placental BDNF can lead to reduced ovarian reserve during fetal development and POI in adulthood. Reduced ovarian reserve was reflected by diminished PGC cells, and varying degrees of decrease in the number of ovarian follicles, especially primordial follicles in cKO(HE) mouse offspring at seven days, one month, two months, and four months after birth, and reduced implantation sites and litter size in cKO(HE) mice. Additionally, in one-month-old mice, there was a reduction in the number of retrieved oocytes after ovulation induction, leading us to speculate that cKO (HE) mice may be insensitive to ovulation-inducing drugs. Ovarian dysfunction can also lead to hormonal instability, affecting the secretion of ovarian hormones. Ovulation-inducing drugs are designed to stimulate the ovaries to produce more eggs, but in cases of ovarian dysfunction, the ovaries may not respond as expected to these medications. Furthermore, due to the limitations in ovarian function, it may impede the normal development of follicles, consequently reducing the effectiveness of the drugs [69, 70]. In our results, there was no statistically significant difference in the number of primordial follicles in the one-month-old mouse ovaries. This may be attributed to our use of ovarian tissues post-ovulation induction for follicle counting. Due to the ovulation induction, a significant portion of ovarian sections contained antral follicles, which affected the counting of primordial follicles.

Cyclin D1 is a key regulator of cell proliferation. Cyclin D1 expression initiates the cell cycle from the G1 phase to the S phase, which promotes cell proliferation [71]. In bovine granulosa cells (GCs), BDNF treatment can activate the AKT signal pathway via TrkB and upregulated cyclin D1 and subsequently promote proliferation and progesterone synthesis by bovine GCs [72]. Cyclin D1 is also an important regulator for PGC proliferation. The research reports that AKT activates the cell cycle in PGCs at early stages [73]. Consistently, the diminished Cyclin D1 expression in PGCs is correlated with the fewer PGCs in cKO(HE) embryos in our study. Insufficient PGCs or ovarian reserve can lead to premature ovarian senescence in mice later in life [74]. Generally, ovarian senescence is a biological process accompanied by a decrease in mitochondrial number, morphological changes in mitochondria, and decreased oxidative phosphorylation in oocytes [75–77]. In our study, we demonstrated not only the decreased ovarian function and reproduction but also altered biomarkers indicating ovarian senescence in young cKO(HE) mice. For example, electron microscope results showed mitochondrial depletion and swelling and apoptotic bodies; transcriptome analysis revealed the decreased oxidative phosphorylation in oocytes of the postnatal-day-7-old cKO(HE) mice.

Transcriptome data discovered that *fgf2* and *hmgb1* genes were dysregulated in cKO(HE) ovaries. Fibroblast growth factor 2 (FGF2), a member of the FGF family, binds with FGF receptors (FGFR) [78–80]. FGF2 promotes follicular development and promote oocyte maturation [81, 82]. FGF2 alliance vascular endothelial growth factor (VEGF) improves revascularization and survival in mouse ovarian [83–85]. The significant down-regulation of *fgf2* in ovaries in postnatal-day-7-old cKO(HE) mice is indicative of reduced follicular formation and ovarian viability in the early postnatal period. The mice had fewer follicles in adults. High mobility group box-1 (HMGB1), an endogenous inflammatory response protein, contributes to inflammatory responses and tissue repair [86, 87]. HMGB1 and HMGB2 are chromatin-associated proteins that belong to the HMG protein family and play a vital role in inflammatory responses and tissue repair [88, 89]. HMGB2-KO mice are subfertile due to reduced numbers of oocytes and follicles, however, HMGB1 was compensatory increased [90]. Recent studies have indicated that there exists a certain level of interaction and regulatory relationship between HMGB1 and BDNF. For instance, in certain inflammatory states, HMGB1 can influence the expression and release of BDNF [91]. Additionally, some research has suggested that the levels of BDNF may be subject to regulation by HMGB1, particularly in neurological disorders and inflammatory processes [91]. Based on this study, we speculate that the increased *hmgb1* expression in ovaries in cKO(HE) mice might also be a compensatory mechanism for aging. Research has found that FGF-2 can increase the replication and transcription of mitochondrial DNA (mtDNA), thereby promoting the process of mitochondrial biogenesis. Additionally, FGF-2 can regulate the activity and expression of mitochondrial respiratory chain complexes, thereby affecting cellular energy metabolism [92, 93]. Experiments suggest that increasing the expression of FGF2 and BDNF may help protect the nervous system from the effects of aging, promoting the survival and maintenance of neurons. Therefore, the reduction of FGF2 accelerates the aging of the ovaries. In addition, studies have shown that increased expression of HMGB1 may trigger inflammatory responses and lead to impairment of mitochondrial function [87, 94, 95]. Mitochondria serve as the primary energy producers within cells, and their proper functioning is crucial for oocytes and granulosa cells within the ovaries. Studies have identified abnormalities in the mitochondria of patients with Premature Ovarian Insufficiency (POI), which may lead to inadequate energy supply, thereby impacting the normal development and function of oocytes [96–98].

The potential mechanism of reduced PGC proliferation in HE female embryos could be through ROS and the stress response in PGCs. Indeed, BDNF deficiency is associated with increased ROS production and cellular stress, which could impact the function of PGCs [99, 100]. Under conditions of BDNF deficiency, cells may fail to effectively activate antioxidant pathways, resulting in increased ROS production and leading to cell damage and death [101]. This is particularly detrimental to PGCs as it can damage lipids, proteins, and DNA within these cells, affecting their viability, proliferation, and capacity to differentiate, ultimately resulting in a reduced number of PGCs [102]. However, the regulation of PGC proliferation involves complex molecular and cellular mechanisms. Some key mechanisms involved: 1) epigenetic modifications, such as DNA methylation and histone modifications, play a crucial role in controlling gene expression. Changes in the epigenetic landscape can affect the proliferation of PGCs. 2) Cell cycle regulation such as cyclin-dependent kinases. 3) Signaling pathways such as BMP, Wnt, and other signaling pathways have been implicated. 4) The microenvironment of Gonadal Niche signals such as growth factors and cytokines. 5) Transcription factors such as Blimp1 and Prdm14. 5) Nutrient and metabolic factors. Therefore, the placental knockout of BDNF could affect PGC proliferation through multiple mechanisms. Firstly, BDNF is a critical cell survival factor, and its reduction could lead to the activation of apoptotic pathways in PGCs, decreasing cell numbers [103]. Secondly, as BDNF can mitigate oxidative stress, its decrease might render PGCs more vulnerable to damage from ROS, thereby reducing their survival rate [101]. Additionally, BDNF may play a role in regulating cell differentiation, so its decreased levels could interfere with the normal differentiation process of PGCs [103]. BDNF is also involved in regulating cell migration, which means its reduction could impede the proper migration of PGCs to the gonads. Lastly, BDNF can influence the microenvironment surrounding PGCs, providing nutritional support and mediating intercellular communication; thus, a decrease in BDNF could disrupt this supportive environment, adversely affecting the survival and proliferation of PGCs [104]. Overall, the reduction in BDNF levels could disrupt the survival, migration, and differentiation of PGCs through the aforementioned mechanisms, leading to a decline in their numbers. These potential mechanisms are hypothesized based on the known functions of BDNF in other systems and cell types, rather than verified results specific to PGCs. We agree with the reviewer that the direct effects of BDNF on PGCs and its role in reproductive biology remain to be further investigated and confirmed. Therefore, our ongoing comprehensive study is designed to investigate these mechanisms.

The RNAseq analysis revealed an aged transcriptome profile in the oocytes of one-month-old mice including a significantly reduced oxidative phosphorylation signal. In aging women, the antioxidant capacity of the body gradually decreases, which reduces the efficiency of oxidative phosphorylation [105, 106]. During oocyte maturation, glucose serves as a crucial energy metabolite for cumulus-oocyte complexes (COCs). However, in the presence of low oocyte energy metabolism, there is a decrease in ATP production and mitochondrial activity. As a result, the oocytes exhibit signs of aging, leading to a reduction in the egg’s fertilization capacity [107]. Therefore, cKO(HE) mice oocytes exhibit signs of aging and lead to a reduction in the egg’s fertilization capacity.

In general, human ovarian function is assessed by AMH, FSH, and menstrual cycle [108–110]. However, in animals, the number of follicles in the ovary is critical for assessing ovarian function [111]. Lower ovarian reserve is also correlated with poor responses to superovulation and a decreased yield of eggs [112]. In addition, the significant increase in FSH expression in the ovaries of four-month-old mice indicates a decline in ovarian function in cKO(HE) mice of the control. PGCs are a specialized group of cells in the early stages of embryonic development, serving as precursors for the formation of reproductive cells (eggs or sperm). Normal development of PGCs is crucial for the subsequent development of gonads, therefore, once the reduction of primordial germ cells occurs, it leads to an insufficient number of follicles in the ovaries, resulting in premature depletion [113].

Furthermore, the overall metabolic health of females and improper neuronal development in the absence of BDNF may also lead to depression and early aging. Indeed, beyond its roles in the neuronal system, BDNF exerts regulatory effects in other parts of the body, particularly in energy metabolism and the endocrine system [114–116]. Future studies are warranted to investigate these potential mechanisms.

In our study, the knockout embryos exhibit lethal phenotypes, which may be attributed to multiple factors. One such cause may be the shutdown of critical mitochondrial functions, such as oxidative phosphorylation, or the accumulation of high levels of reactive oxygen species (ROS) within cells, which can lead to cellular necrosis or apoptosis. In our study, we only observed a small portion of resorptions of HO embryos (*n* = 3). Based on Fig. 1c (3.4% HO embryo), we suspected the lethal phenotype is most likely to be fertilization and/or implantation deficiencies (gaps but no visible resorptions) of HO embryos. From our analysis of the morphology of dead embryos during gestation, the lack of BDNF in the placenta may cause developmental defects and impaired physiological and neurological function. The specific mechanisms, however, require further investigation.

### Limitations

The limitation of our study is that we cannot determine whether placenta function affects fetal ovarian development independent from BDNF signal in the fetal compartment. It has been shown that BDNF affects placental development by promoting the proliferation and survival of trophoblast cells [57]. Therefore, placental-specific knockdown of BDNF may disrupt placental function in cKO(HE) mice. Further studies are warranted to investigate the link between these mice’s placental development and fetal programming.

## Conclusion

Here, our study demonstrated that decreased placental BDNF leads to adult POI by decreasing the proliferation of early primordial germ cells and establishing ovarian reserve in mice. Our study provides new insight into the etiology of POI and indicates that it is critical to identify potential risks and intervention strategies for POI during early life.

### Supplementary Information

Supplementary Material 1.

## Data Availability

No datasets were generated or analysed during the current study.
