# Supplementary material for: Depletion of placental brain-derived neurotrophic factor (BDNF) is attributed to premature ovarian insufficiency (POI) in mice offspring
Source: J Ovarian Res. 2024 Jul 9;17:141. doi: 10.1186/s13048-024-01467-4 (PMC11232340; doi:10.1186/s13048-024-01467-4)

**supplementary materials**

**Supplementary Table 1.** Primers for *hmgb1*, *fgf2* and *gapdh*

| **Gene** | Forward sequence (5'-3') | Reverse sequence (5'-3') |
| --- | --- | --- |
| *gapdh* | AAGAAGGTGGTGAAGCAGG | GAAGGTGGAAGAGTGGGAGT |
| *hmgb1* | CCCAGCGAAGGCTATCACA | TTCATAAAGGGACAAACCACAG |
| *fgf2* | TTGCTATGAAGGAAGATGGACG | CCCAGTTCGTTTCAGTGCC |
| *pcdhb21* | GCTAGTCAAGGACAATGGCG | ACAGACAAGAGGAAGAGCGAAG |

**Supplementary Table 2.** *hmgb1*, *pcdhb21* and *fgf2* expression

| **Gene ID** | gene_name | Description | FC | |
| --- | --- | --- | --- | --- |
|  |  |  | RNAseq | realtime |
| **ENSMUSG00000066551** | *hmgb1* | high mobility group box 1 | 2.5 | 2.02 |
| **ENSMUSG00000037225** | *fgf2** | fibroblast growth factor 2 | -4.47 | -9.29 |
| **ENSMUSG00000044022** | *pcdhb21** | protocadherin beta 21 | 12.18 | 2.05 |

**Supplementary Table 3.** List of top 10 differentially expressed genes between control and cKO(HE) mice, ranked according to p-values

| **Gene Name** | Annotation | Fold Change (cKO/Control) | P value |
| --- | --- | --- | --- |
| ***rpl37rt*** | ribosomal protein L37, retrotransposed | 203.04 | 3.67454E-37 |
| ***gm4350*** | predicted gene 4350 | 13.28 | 7.38054E-14 |
| ***pcna-ps2*** | proliferating cell nuclear antigen pseudogene 2 | 19.62 | 6.44209E-12 |
| ***pakap*** | paralemmin A kinase anchor protein | 100.25 | 1.05204E-08 |
| ***gm17535*** | predicted gene, 17535 | 7278.59 | 4.86076E-07 |
| ***pla2g4e*** | phospholipase A2, group IVE | 9.50 | 1.47861E-06 |
| ***gm10800*** | predicted gene 10800 | 516.01 | 9.32042E-06 |
| ***duox2*** | dual oxidase 2 | 46.78 | 1.19778E-05 |
| ***gm10722*** | predicted gene 10722 | 302.84 | 1.18436E-05 |
| ***lrrc14*** | leucine rich repeat containing 14 | 5.538 | 2.11363E-05 |
| ***ugcg*** | UDP-glucose ceramide glucosyltransferase | -8.73 | 1.23E-21 |
| ***gm37915*** | predicted gene, 37915 | -154.89 | 2.48E-17 |
| ***uqcr11*** | ubiquinol-cytochrome c reductase, complex III subunit XI | -12.43 | 6.32E-17 |
| ***uba52*** | ubiquitin A-52 residue ribosomal protein fusion product 1 | -13.16 | 2.02E-15 |
| ***rpl41*** | ribosomal protein L41 | -9.30 | 1.64E-10 |
| ***ndufa11*** | NADH:ubiquinone oxidoreductase subunit A11 | -5.42 | 4.36E-08 |
| ***gm1673*** | predicted gene 1673 | -4.81 | 5.98E-08 |
| ***gm10076*** | predicted gene 10076 | -12.34 | 9.64E-08 |
| ***samhd1*** | SAM domain and HD domain, 1 | -3.80 | 1.06E-07 |
| ***gm6166*** | predicted gene 6166 | -53.57 | 7.84E-06 |

**Supplementary Table 4**. List of top 5 genes of arginine and proline metabolism and top 5 genes of oxidative phosphorylation

| **Top 5 genes of arginine and proline metabolism** | | | |
| --- | --- | --- | --- |
| **Gene Name** | **Annotation** | **Fold Change (cKO/Control)** | **P value** |
| *azin2* | antizyme inhibitor 2 | 30.94983 | 0.011739 |
| *arg1* | arginase, liver | 24.98003 | 0.029765 |
| *aoc1* | amine oxidase, copper-containing 1 | 17.32677 | 0.017056 |
| *prodh* | proline dehydrogenase | 5.370391 | 0.0309 |
| *smox* | spermine oxidase | 2.340781 | 0.023342 |
| **List of top 5 genes of oxidative phosphorylation** | | | |
| *uqcr11* | ubiquinol-cytochrome c reductase, complex III subunit XI | -12.43042 | 6.32E-17 |
| *ndufs8* | NADH:ubiquinone oxidoreductase core subunit S8 | -10.01772 | 0.044662 |
| *cox11* | cytochrome c oxidase assembly protein 11, copper chaperone | -8.014056 | 0.045314 |
| *ndufa7* | NADH:ubiquinone oxidoreductase subunit A7 | -5.472158 | 0.04254 |
| *ndufa11* | NADH:ubiquinone oxidoreductase subunit A11 | -5.423126 | 4.36E-08 |

**Supplementary Figure 1**. The breeding strategy to generate placenta BDNF knockout mice (A) and genotyping (B).


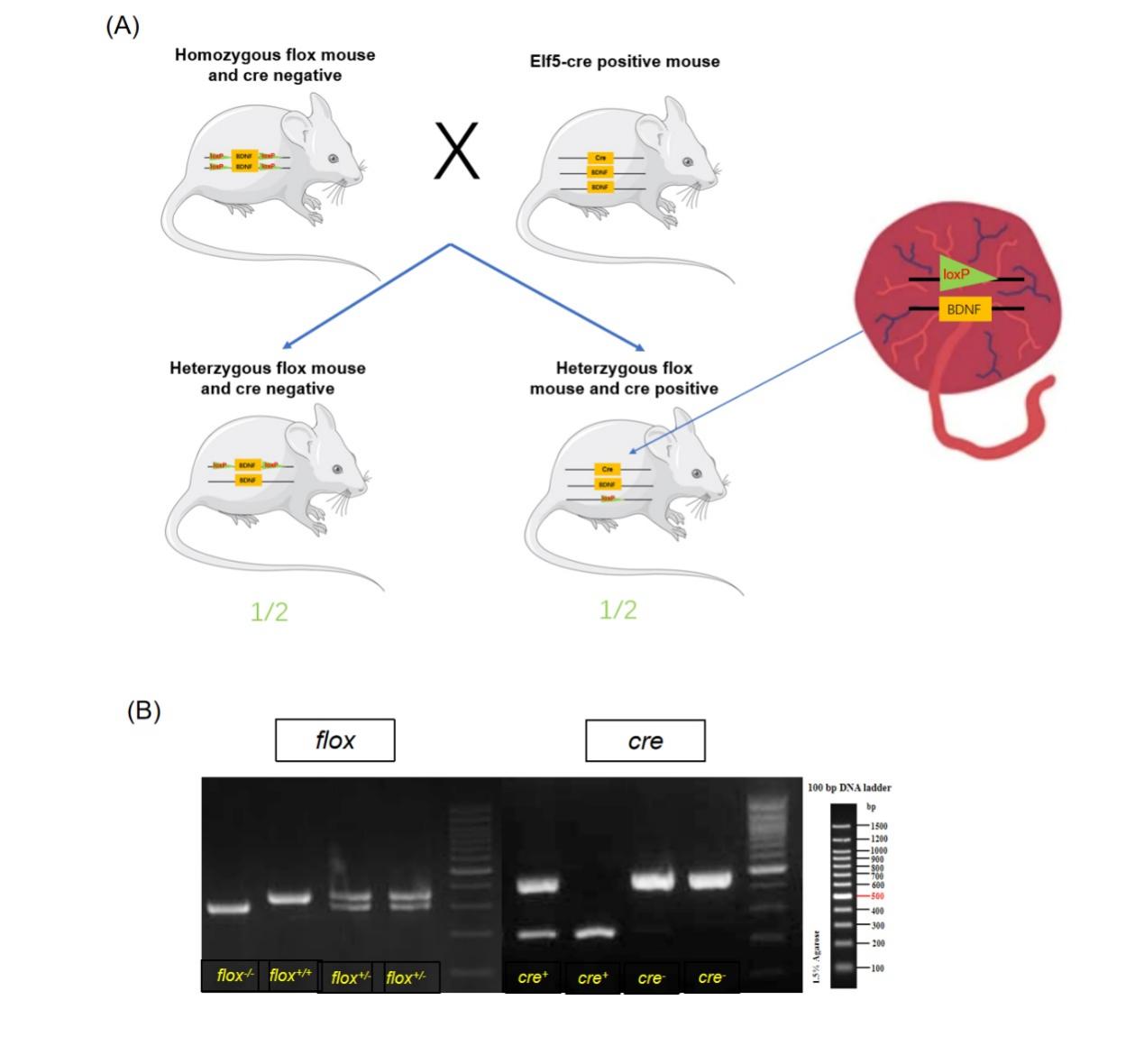


**Supplementary Figure 2**. Full Western blots of BDNF.


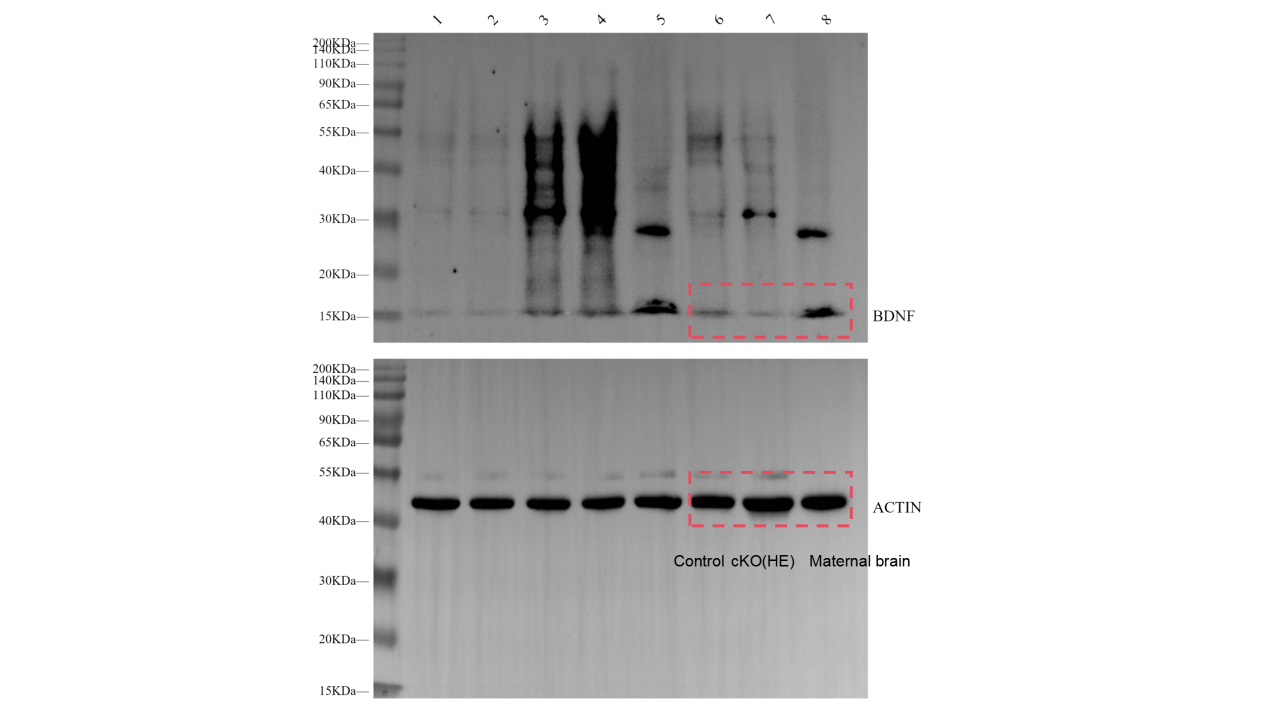

Supplement: Supplementary file 1 — Supplementary Material 1. [file 13048_2024_1467_MOESM1_ESM.docx]
